# Chloride Activated Halophilic *α*-Amylase from *Marinobacter* sp. EMB8: Production Optimization and Nanoimmobilization for Efficient Starch Hydrolysis

**DOI:** 10.1155/2015/859485

**Published:** 2015-01-18

**Authors:** Sumit Kumar, S. K. Khare

**Affiliations:** Department of Chemistry, Indian Institute of Technology Delhi, Hauz Khas, New Delhi 110016, India

## Abstract

Halophiles have been perceived as potential source of novel enzymes in recent years. The interest emanates from their ability to catalyze efficiently under high salt and organic solvents. Present work encompasses production optimization and nanoimmobilization of an *α*-amylase from moderately halophilic *Marinobacter* sp. EMB8. Media ingredients and culture conditions were optimized by “one-at-a-time approach.” Starch was found to be the best carbon source at 5% (w/v) concentration. Glucose acted as catabolic repressor for amylase production. Salt proved critical for amylase production and maximum production was attained at 5% (w/v) NaCl. Optimization of various culture parameters resulted in 48.0 IU/mL amylase production, a 12-fold increase over that of unoptimized condition (4.0 IU/mL). *α*-Amylase was immobilized on 3-aminopropyl functionalized silica nanoparticles using glutaraldehyde as cross-linking agent. Optimization of various parameters resulted in 96% immobilization efficiency. Starch hydrolyzing efficiency of immobilized enzyme was comparatively better. Immobilized *α*-amylase retained 75% of its activity after 5th cycle of repeated use.

## 1. Introduction

*α*-Amylases are important class of industrial enzyme finding wide scale applications in food, textile, paper, detergent, analytical chemistry, beverage, and pharmaceutical industry. Demand for *α*-amylase is projected to further increase in coming years due to its use in diverse industrial sectors [1–5]. *α*-Amylases from wide range of sources with distinct characteristics are available. Yet search continues for novel *α*-amylase to increase the realm of processes where it can be used. In this context, isolation and screening of extremophilic organisms for *α*-amylase of desired trait is a contemporary research area. Halophiles, a class of extremophiles growing under saline conditions, offer source of enzymes which not only are salt stable but also can function under conditions of alkaline pH, high temperature, and low water activity [6–8]. The use of halophilic *α*-amylase in bioprocesses presents the advantage to obtain optimal activities at high salt concentrations. Halophilic *α*-amylases also might be particularly resistant to organic solvents because they work under condition where water activity is low.

Halophilic amylases have been reported from extreme halophiles such as* Natronococcus amylolyticus* [9];* Haloferax mediterranei* [10];* Haloarcula hispanica* [11].* Halomonas meridiana* [12];* Chromohalobacter* sp. TVSP 101 [13];* Nesterenkonia* sp. strain F [14] are moderately halophilic bacteria reported to produce halophilic amylase. In general the production level of amylases in halophiles is very low. Conditions have been optimized in case of* Halomonas meridiana* [12],* Halobacillus* sp. strain MA-2 [15], and* Bacillus* sp. strain TSCVKK [16] for enhancing the yield, yet maximum 3.2 U/mL could be attained.

Another aspect covered in this study is immobilization of *α*-amylase. The immobilization of enzymes on solid support offers several advantages over the free enzyme, including easy recovery from the reaction medium, reusability, possibility of operation in continuous reactors, enhanced stability, and catalytic efficiency [17]. There have been many reports on immobilization of *α*-amylase. Some examples involve reactive polymer films [18], magnetic nanoparticles [19], mesoporous silica thin films [20], and adsorption on zirconia [21]. In recent years, nanostructured materials such as nanoporous media, nanofibers, nanotubes, and nanoparticles have emerged as amazingly effective enzyme support/matrix [22–25]. They provide the highest possible surface area for immobilization, enabling very high loading of enzyme on the support. This results in surprisingly high enzyme activities per unit volume [26].

Amylase producer halophilic bacteria* Marinobacter* sp. EMB8 was isolated during screening of Indian saline habitats [27]. The *α*-amylase was purified and found to be salt and solvent stable. It was used for synthesis of industrially useful maltooligosaccharides [28]. With the above viewpoints, production optimization for competitive yields and immobilization for efficient application of *α*-amylase from* Marinobacter* sp. EMB8 were attempted. Bacterial growth and enzyme production are greatly influenced by the nutritional factors (carbon and nitrogen sources, metal ions, etc.) and physical factors (pH, temperature, inoculation volume, and incubation time). A systematic investigation for effect of these factors on *α*-amylase production by* Marinobacter* sp. EMB8 has been undertaken in the present study. We herein also attempted to immobilize the halophilic* Marinobacter* sp. *α*-amylase on silica nanoparticles to obtain an active, stable, reusable preparation for effective starch hydrolysis.

## 2. Materials and Methods

### 2.1. Materials

3-Aminopropyl functionalized silica nanoparticles and betaine were product of Sigma Chemical Company (St. Louis, MO, USA). Soluble starch and glutaraldehyde were obtained from Merck Specialties Pvt. Ltd. (Mumbai, India). Bovine serum albumin (BSA) was purchased from Sisco Research Laboratories Pvt. Ltd. (Mumbai, India). The media components were purchased from Hi-Media Laboratories (Mumbai, India). All other chemicals used were of analytical grade.

### 2.2. Microorganism and Culture Conditions

*Marinobacter* sp. EMB8 used in present study was isolated from the Indian sea coast of Kozhikode (Kerala, 11°25′N 75°77′E) during screening of halophilic bacteria for industrially important hydrolases. Amylolytic activity of the culture was screened on starch nutrient agar plates [27]. The isolate was stored at 4°C and subcultured on nutrient agar medium containing starch (10.0 g/L) at 15-day intervals.

### 2.3. Amylase Assay

Amylase was assayed following the method of Bernfeld [29] using starch as substrate. One mL of reaction mixture contained 500 *μ*L of soluble starch (2%, w/v) in sodium phosphate buffer (50 mM, pH 7.0) containing 1% (w/v) NaCl and 500 *μ*L enzyme. Reaction was carried out for 20 minutes at 45°C. The reducing sugar released by amylolytic activity was measured by the reduction of 3, 5-dinitrosalicylic acid (DNSA). One unit of amylase activity was defined as the amount of enzyme releasing 1 *μ*mol of maltose equivalent per minute from soluble starch under assay conditions. Protein concentration was estimated by dye binding method [30], using BSA as a standard protein.

### 2.4. Optimization of Culture Conditions

Mother culture was prepared by inoculating a loopful of* Marinobacter* sp. EMB8 from the slant, into 25 mL of medium (A) containing (g/L) starch, 10.0; peptone, 5.0; yeast extract, 5.0, with NaCl, 50.0; pH 7.0. Overnight grown culture (OD ~1.0) was used as inoculum. Media optimization for amylase production was carried out by “one-at-a-time approach” wherein single parameter was changed at a time while keeping others at a constant level. In order to see the effects of various nutritional and physical factors on amylase production, medium (A) was used as basal. Various carbon, nitrogen sources, and metal ions were varied, one at time, in the media. One percent of the mother culture was seeded as inoculum into 50 mL medium contained in 250 mL Erlenmeyer flask. The incubation was carried out at 35°C and 200 rpm for 72 h. The growth was monitored by recording the absorbance of the culture at 660 nm against uninoculated culture as blank.

For checking the amylase activity, cells were harvested after 72 h by centrifugation at 10,000 ×g for 10 min at 4°C. The cell-free supernatant filtered through a 0.22 *μ*m cellulose acetate membrane (Millipore Corporation, MA, USA) was assayed. Effect of different parameters was monitored on the growth (*A*_660 nm_) and amylase activity.

#### 2.4.1. Carbon Source

Different carbon sources (1%, w/v), namely, glucose, fructose, maltose, lactose, sucrose, cellulose, dextrin, and starch, were supplemented in the medium containing (g/L) peptone, 5.0; yeast extract, 5.0; NaCl, 50.0. Medium was inoculated with 1% (v/v) mother culture and incubation was carried out at 35°C for 72 h at 200 rpm shaking.

Effect of concentration of starch, the best utilized carbon source, was tested by incorporating it in different concentrations ranging from 0 to 10% (w/v). Other culture conditions were kept the same. Growth and amylase production were monitored as described above.

#### 2.4.2. Nitrogen Source

The effect of various inorganic and complex nitrogen sources was studied by replacing yeast extract and peptone in the medium containing (g/L) starch, 50.0; K_2_HPO_4_, 0.87; MgSO_4_ *·*7H_2_O, 6.2; KCl, 0.75; NaCl, 50.0, with other nitrogen sources, namely, (NH_4_)_2_SO_4_, NH_4_Cl, urea, gelatin, casein enzyme hydrolysate, yeast extract, peptone, tryptone, combination of yeast extract and peptone, and corn steep liquor. All the nitrogen sources were incorporated at 1% (w/v) nitrogen content. Culture samples were processed for amylase assay and growth measurements similarly.

Casein enzyme hydrolysate was found to be utilized best among various nitrogen sources tested. In order to investigate the influence of casein enzyme hydrolysate concentration, its concentration was varied (0.5–3.0%, w/v) in the medium.

#### 2.4.3. Salt and Metal Ions

The strain was grown in varying concentrations (0–20%, w/v) of NaCl in the medium containing (g/L) starch, 50.0; casein enzyme hydrolysate, 10.0; K_2_HPO_4_, 0.87; MgSO_4_ *·*7H_2_O, 6.2; KCl, 0.75, with pH 7.0 seeded with 1% (v/v) inoculum at 35°C and 200 rpm. For evaluation of effect of metals, a range of metal ions at concentration 0.1% (w/v) was added into medium containing (g/L) starch, 50.0; casein enzyme hydrolysate, 10.0; NaCl, 50.0, to see the outcome on growth and amylase production.

#### 2.4.4. Effect of pH of the Medium and Incubation Temperature

The influence of pH on growth and amylase production was monitored by adjusting the pH of the optimized medium (designated as B) (g/L): starch, 50.0; casein enzyme hydrolysate, 10.0; K_2_HPO_4_, 0.87; MgSO_4_ *·*7H_2_O, 6.2; KCl, 0.75; NaCl, 50.0 to different values, ranging from 6.0 to 10.0.

The effect of temperature was studied by incubating culture flasks at various temperatures ranging from 25°C to 45°C. The medium B adjusted to pH 7.0 was used and other culture conditions were essentially the same.

#### 2.4.5. Effect of Inoculum Size and Shaking Condition

Varying inoculum sizes (ranging from 0.5 to 5.0%, v/v) of overnight grown culture were used. Other conditions were kept as optimized. The medium B shaking speed during incubation was varied from static to 250 rpm. Other conditions were kept as those optimized, namely, media B adjusted to pH 7.0, inoculum 1% (v/v), and incubation at 35°C for 72 h.

#### 2.4.6. Cell Growth and Amylase Production under Optimized Conditions

Amylase production was carried out under finally optimized conditions, namely, medium containing (g/L) starch, 50.0; casein enzyme hydrolysate, 10.0; K_2_HPO_4_, 0.87; MgSO_4_ *·*7H_2_O, 6.2; NaCl, 50.0, with pH 7.0 seeded with 1% (v/v) inoculum at 35°C and 200 rpm. Samples were withdrawn at different time intervals to measure the bacterial growth and amylase production as described above.

### 2.5. Effect of Chloride Ions on *α*-Amylase Activity

Effect of chloride ions on *α*-amylase activity was checked by adding 1% (w/v) of various anions (fluoride, chloride, bromide, iodide, nitrate, azide, and acetate) of sodium and potassium in the assay mixture.

### 2.6. Immobilization of *α*-Amylase

Covalent coupling of *α*-amylase on the functionalized silica (Sigma Cat. number 660442, average particle size ~100 nm) was performed in a sodium phosphate buffer (50 mM, pH 7.0) using glutaraldehyde as a cross-linking agent. For the immobilization, the NH_2_ groups of silica (15 mg/mL in buffer) were activated with glutaraldehyde (2.5%, v/v). The mixture was incubated at 25°C for 2 h with continuous shaking at 200 rpm. It was further washed eight times with buffer to remove unbound glutaraldehyde. The activated nanosilica was incubated with 1 mL of *α*-amylase (90 IU; in 50 mM sodium phosphate buffer, pH 7.0) for 2 h at 25°C under constant shaking at 200 rpm. The unbound enzyme was removed as supernatant by centrifuging at 8000 ×g for 10 min. The pellet which consisted of nanosilica containing immobilized *α*-amylase was washed by buffer until protein free supernatant was obtained. The immobilized *α*-amylase was finally suspended in sodium phosphate buffer (50 mM, pH 7.0) and stored at 4°C. Immobilization efficiency was calculated as follows:

(1) Immobilization  efficiency =Total  activity  of  the  immobilized  α-amylaseTotal  activity  of  the  free  α-amylase  ×100.

### 2.7. Optimization of Immobilization Conditions

Conditions were optimized to attain maximum immobilization efficiency, by varying one-at-a-time: glutaraldehyde concentration (0.25–2.5%, v/v); nanoparticle concentration (7.5–45.0 mg); enzyme loading (45–180 IU); and effect of protectants maltose, starch, betaine, and BSA (0.2–1.0%, w/v).

### 2.8. Starch Hydrolysis

The starch solution was prepared as 2.0% (w/v) in sodium phosphate buffer (50 mM, pH 7.0) containing 1% (w/v) NaCl. For hydrolysis in the batch process, 20 mL of starch solution was mixed with soluble or immobilized *α*-amylase (2 IU/mL) in 150 mL flasks. The hydrolysis was carried out at 45°C with continuous shaking at 200 rpm for 8 h. The aliquots were taken out at various time intervals and the formation of the reducing sugar was quantitatively determined by the DNSA reagent.

### 2.9. Reusability of the Immobilized *α*-Amylase

The starch hydrolyzing activity of the immobilized enzyme was evaluated by reusing the immobilized preparation after each use. After each cycle, the bound enzyme was separated by centrifugation, washed, and stored in the same buffer. It was then used for next starch hydrolysis cycle as described in previous section. The *α*-amylase activity was determined each time. The activity recorded in the first cycle was taken as 100% activity, for calculating the residual activity after each successive usage.

All the experiments were done in triplicate and the variation was within ±5%.

## 3. Results and Discussion

Halophiles have been perceived as a potential source of industrially useful enzymes endowed with unique stabilities [31–33].* Marinobacter* sp. EMB8 used in present study was isolated by us from Kozhikode sea water, India. The isolate was Gram-negative rod and grew well at high salt concentrations (1–20%, w/v) and pH range of 6.0–9.0. On the basis of salt requirement for growth, it could be placed under moderately halophilic bacterium according to Kushner's [34] classification. Identification of isolate by 16S rDNA analysis related it to genus* Marinobacter*. The sequence has been submitted in GenBank, NCBI, USA, with accession number GU059908.* Marinobacter* sp. EMB8 culture has been deposited in Microbial Type Culture Collection and Gene Bank (MTCC), Chandigarh, India, with MTCC number 12013. The isolate secretes amylase as observed by zone of hydrolysis on starch agar plate. The amylase production was confirmed by growing the cells in starch containing medium. The conditions were optimized further for maximum production of amylase.

### 3.1. Optimization of Amylase Production

#### 3.1.1. Effect of Carbon Source

**Figure 1:**
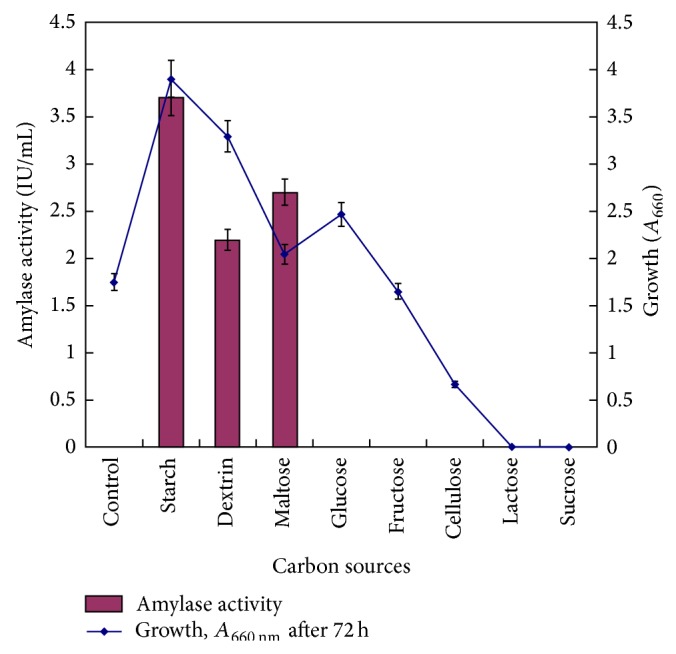
Effect of carbon sources on growth and amylase production. Medium (g/L: peptone, 5.0; yeast extract, 5.0; NaCl, 50.0; pH 7.0) was supplemented with different carbon sources (1.0%, w/v). Control was without any carbon source. Medium was inoculated with 1% mother culture and incubation was carried out at 35°C for 72 h at 200 rpm shaking.

Different carbon sources at 1% (w/v) concentrations were used to see their effect on growth and amylase production. Results are shown in Figure 1. Bacterial growth was supported by all the sugars except sucrose and lactose. However, amylase was produced only with starch, dextrin, and maltose. Starch was found to be the best carbon source for amylase production. No amylase activity was detected in case of glucose, fructose, sucrose, lactose, and cellulose. Amylase production among halophiles is generally inducible and needs suitable inducers such as starch and dextrin in the medium.

Starch has been observed as best inducer for amylase production in case of* Halobacterium halobium* [35] and* Halomonas meridiana* [12]. Glucose possibly acted as catabolic repressor for amylase production. This was further checked by adding glucose in the medium with starch. Addition of glucose caused delayed amylase production and decreased the yield by 46%. Glucose as repressor for amylase production has been previously reported in* Micrococcus* sp. [36],* Natronococcus* sp. strain Ah-36 [37], and* Halomonas meridiana* [12].

Concentration of starch was varied in medium to achieve optimum production. Amylase production increased as a function of starch concentration reaching maximum 15 IU/mL at 5% (w/v). No amylase production was detected in absence of starch. Starch at 5% (w/v) concentration was used as carbon source throughout further studies.

#### 3.1.2. Effect of Nitrogen Source

**Figure 2:**
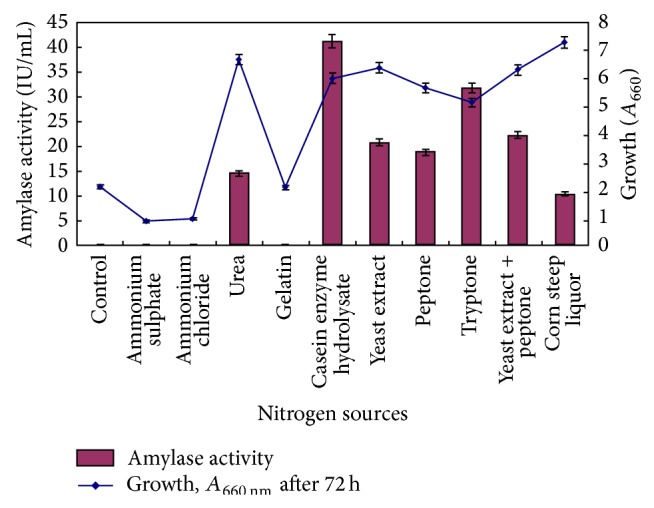
Effect of nitrogen sources on growth and amylase production. Medium (g/L: starch, 50.0; K_2_HPO_4_, 0.87; MgSO_4_ *·*7H_2_O, 6.2; KCl, 0.75; NaCl, 50.0; pH 7.0) was supplemented with different nitrogen sources at concentration so as to provide 1% (w/v) nitrogen. Medium was inoculated with 1% mother culture and incubation was carried out at 35°C for 72 h at 200 rpm shaking.

Nitrogen sources were varied (keeping nitrogen content 1%, w/v) to see the effect on growth and amylase production (Figure 2). Complex nitrogen sources were found to be better. Amongst these, casein enzyme hydrolysate was found to be the best nitrogen source for amylase production.

Since the highest production was obtained with casein enzyme hydrolysate, the effect of its concentration on amylase production was further optimized. Amylase production was optimum in the medium with casein enzyme hydrolysate concentrations 1 and 1.5% (w/v). Casein enzyme hydrolysate concentration beyond this led to decrease in amylase production. Casein enzyme hydrolysate at 1% (w/v) concentration was considered best and used for further optimization.

Peptone in case of* Halobacterium halobium* [35] and* Halorubrum xinjiangense* [38]; combination of yeast extract and tryptone in case of* Bacillus* sp. strain TSCVKK [16]; tryptone in case of* Chromohalobacter* sp. TVSP101 [13] are some reports of best nitrogen source for amylase production. No nitrogen source can be termed as universally good but organic nitrogen source works better for amylase production in halophiles.

#### 3.1.3. Effect of Salt (NaCl) and Metal Ions

**Figure 3:**
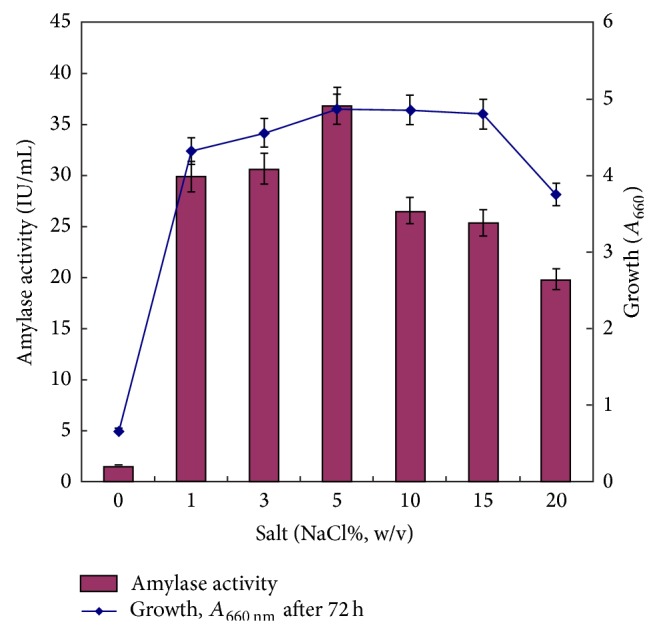
Effect of salt (NaCl) concentration on growth and amylase production. Experimental conditions were kept as optimized above. Medium (g/L: starch, 50.0; casein enzyme hydrolysate, 10.0; K_2_HPO_4_, 0.87; MgSO_4_ *·*7H_2_O, 6.2; KCl, 0.75; pH 7.0) was supplemented with varying concentrations of NaCl. Medium was inoculated with 1% mother culture and incubation was carried out at 35°C for 72 h at 200 rpm shaking.

Salt proved critical as amylase production was severely affected in absence of salt. Optimum production was observed in presence of 5% (w/v) NaCl (Figure 3). Amylase production was even observed at high salt concentration of 20% (w/v) NaCl, confirming halophilic nature of bacteria as well as amylase produced by it. Salt is vital for growth and amylase production in halophiles and preferred salt is generally sodium chloride. Optimum concentration of salt varies from 5 to 25% (w/v) for maximum production. Optimized salt (NaCl) concentrations in some of production studies were 5% for* Halomonas meridiana* [12]; 10% for* Bacillus* sp. strain TSCVKK [16]; 20% for* Chromohalobacter* sp. TVSP101 [13]; 25% for* Halobacterium halobium* [35].

A range of metal ions at concentration 0.1% (w/v) were tested to see their effect on growth and amylase production. Magnesium sulphate in combination with K_2_HPO_4_ was found to be best for amylase production. No growth and amylase production were observed in case of CuSO_4_, CoSO_4_, and HgCl_2_. Amylase production was increased by calcium chloride in* Bacillus* sp. strain TSCVKK [16] and* Chromohalobacter* sp. TVSP 101 [13]. In a different study, zinc sulphate stimulated amylase production in* Halobacterium halobium* [35]. Amylase production in* Halobacillus* sp. strain MA-2 was best in presence of sodium arsenate, while copper sulphate decreased and lead nitrate did not affect the production [15].

#### 3.1.4. Effect of pH and Temperature of the Medium

Amylase production was observed in the pH range of 6.0–9.0. Optimum production was observed at pH 7.0 and 7.5. Growth as well as enzyme production was observed at alkaline pH suggesting the haloalkaliphilic nature of bacteria.

Different temperatures in range of 25–45°C were tried to observe effect on growth and amylase production. Optimum enzyme production level was attained at 35°C. Among other halophiles,* Halobacillus* sp. produced maximum amylase at pH 7.8 and temperature of 30°C [15]. In case of* Bacillus* sp. strain TSCVKK, amylase production was maximum at 30°C and pH 8.0 [16]. For* Chromohalobacter* sp. TVSP101 optimum conditions were pH 9.0 and 37°C [13]. In general, slightly alkaline pH and temperature about 30–37°C favor better amylase production in halophiles.

#### 3.1.5. Effect of Inoculum Size and Shaking Speed

Comparable amylase production was observed for inoculum size in range of 0.5 to 5% (v/v) with optimum being at 1% (v/v).

Aeration is very critical for growth and metabolism of aerobic microbes. In order to check the effect of shaking speed on biomass and amylase production, the* Marinobacter* sp. was grown under optimized conditions with varying shaking speeds. Amylase production increased with increasing shaking speed, optimum achieved at 200 rpm. Interestingly, growth and amylase production were also observed under static condition. Effect of shaking speed and inoculum size on amylase production has been less investigated in halophilic bacteria.* Halobacillus* sp. was found to produce maximum amylase at shaking speed of 200 rpm [15].

#### 3.1.6. Growth of* Marinobacter* sp. and Amylase Production under Optimized Culture Conditions

**Figure 4:**
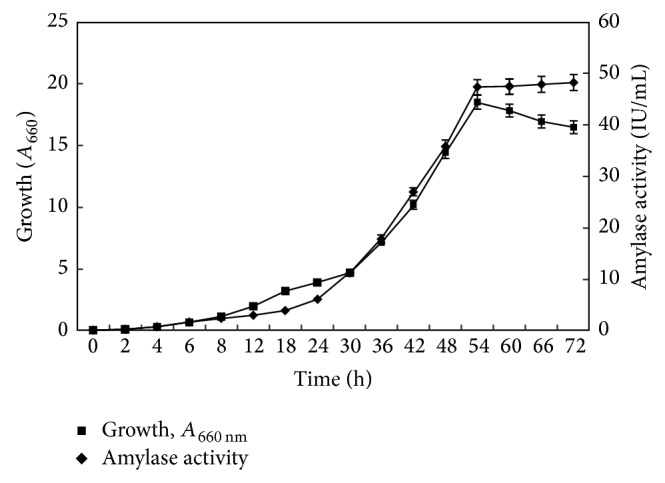
Growth and amylase production from* Marinobacter* sp. EMB8. The bacterium was grown in optimized medium as described in Section 2. Samples were aseptically withdrawn at various time intervals and growth recorded as *A*_660 nm_. Amylase activity was determined in cell-free supernatant.

Amylase production by* Marinobacter* sp. EMB8 was carried out under finally optimized culture conditions. Amylase production was growth-dependent reaching maximum in 54 h as illustrated in Figure 4. In halophiles, amylase production is usually growth-dependent starting in exponential phase and reaching maximum in stationary phase. Similar growth-dependent amylase production has been observed in* Halomonas meridiana* [12],* Bacillus* sp. strain TSCVKK [16], and* Nesterenkonia* sp. strain F [14].

Media optimization for efficient amylase production led to twelvefold increase in production over unoptimized conditions. Amylase production level was quite good as compared to previously reported levels among halophiles.

Present study is important from the viewpoint of low amylase production level secreted in halophiles. Significant increase was attained for* Marinobacter* sp. EMB8. Study also revealed influence of various culture conditions on halophilic amylase production. The starch concentration, nitrogen source, and metal ions were critical and caused significant increase in yield.

### 3.2. Effect of Chloride Ions on *α*-Amylase Activity

**Figure 5:**
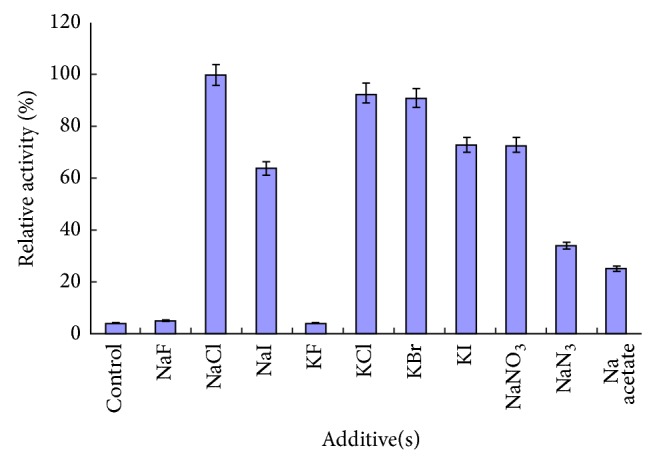
Effect of varying anions on* Marinobacter* sp. *α*-amylase activity: chloride ion activation. Appropriate dilution of *α*-amylase was assayed as per standard procedure. NaCl (1%, w/v) was replaced with different salts in assay mixture. Control sample had no salt in assay mixture. *α*-Amylase activity in presence of NaCl was taken as 100%.

Increasing evidence is gathering to support that anions especially chloride play a critical role in activating *α*-amylases [39]. To validate this,* Marinobacter* sp. *α*-amylase activity was checked in presence of sodium and potassium salt of varying anionic groups. Their effect on activity is illustrated in Figure 5.

The *α*-amylase was found to be activated by chloride ions. Bromide and iodide ions which are of comparable size to chloride ions also acted as activators but to a lesser extent. Fluoride ions which are of smaller size were not able to do so. Other anions comparable to chloride ions in size such as acetate, nitrate, and azide also activated *α*-amylase to varying degrees. Chloride ions are reported to act as allosteric activator in case of human as well as halophilic *α*-amylases such as* Pseudoalteromonas haloplanktis*. Binding of chloride ions leads to interaction with catalytic residues and ultimately activation of *α*-amylase activity [40, 41].

### 3.3. Immobilization of *α*-Amylase on Silica Nanoparticle

In order to explore viable application of this enzyme, it was thought to use in immobilized form. It was immobilized on silica nanoparticles. The choice of silica as a matrix for immobilization was dictated by the fact that they are chemically inert and biocompatible. Also, silica nanoparticles can be easily functionalized [42, 43]. The immobilization was carried out by using two different approaches, that is, (i) simple adsorption and (ii) covalent coupling by using glutaraldehyde. The results are shown in Table 1.

The adsorption method was not very effective as only 12% immobilization efficiency was attained and most of the activity came out in washing as unbound enzyme. This was not surprising as *α*-amylase is loosely bound through weak forces and washing leads to enzyme leaching in the washing fractions.

Covalent coupling placed the enzyme firmly on the silica nanoparticles and about 3.5-fold more immobilization efficiency was attained at 2.5% (v/v) glutaraldehyde concentration. The optimum immobilization efficiency was enhanced by 1.0- to 2.0-fold by glutaraldehyde functionalization in case of cholesterol oxidase immobilization on silica-coated magnetic nanoparticles [43]. Glutaraldehyde treatment of immobilization support leads to availability of free aldehyde groups. The free amino group present on enzyme can readily couple with aldehyde to form imine [44]. The covalent coupling using glutaraldehyde fixes enzyme strongly to the support preventing the chances of leaching. Considering its versatility, glutaraldehyde is very often used for functionalization and coupling of nanoparticles. Soleimani et al. [45] attained 79% immobilization efficiency of *α*-amylase (termamylaze) on silica nanoparticles. In another study* Bacillus amyloliquefaciens* *α*-amylase immobilized on polyaniline-assisted silver nanoparticles through glutaraldehyde coupling retained 83% of its activity [24].

### 3.4. Optimization of Immobilization Conditions

**Figure 6:**
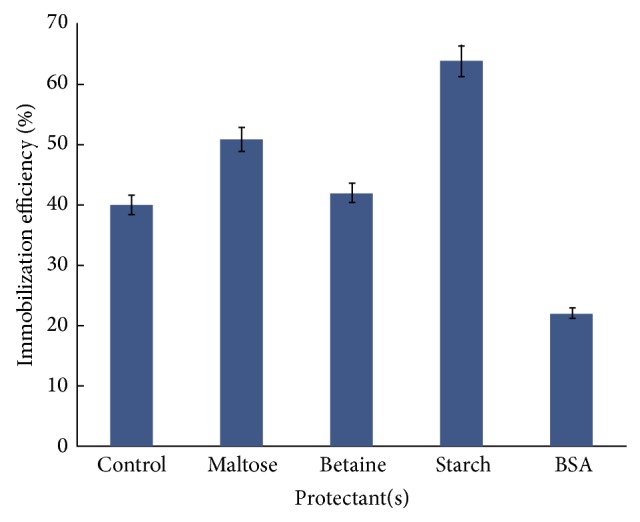
Protective effects of additives on *α*-amylase immobilization efficiency. Different additives at 0.2% (w/v, enzyme nanoparticle mixture) were added along with *α*-amylase (100 IU in 1.0 mL 50 mM sodium phosphate buffer, pH 7.0) to glutaraldehyde functionalized silica nanoparticles (15 mg). In control, *α*-amylase was added to glutaraldehyde functionalized silica nanoparticles without any protectant. Immobilization was carried out as described in Section 2.

Immobilization was carried out in presence of substrate starch, product maltose, neutral protein feeder BSA, and halophilic protein protectant betaine to protect the structure of *α*-amylase and subsequent activity loss during immobilization. Results in Figure 6 show that maltose and starch protected the *α*-amylase and helped 11% and 24% increase in immobilization efficiency, as compared to that of control (in absence of any protectant).

This effect can be attributed to the fact that starch as substrate may have bound to active site and protected the part from denaturation during glutaraldehyde coupling. Maltose is also a product, albeit less effective. Varying concentration of starch as protectant showed that 0.5% (w/v) was sufficient to give maximum protective effect.

The amount of nanoparticles used for immobilization is critical because the immobilization efficiency will largely depend on the available surface area. In our case, 15 mg nanoparticle was found to be optimum. Finally the conditions of immobilization were optimized in terms of optimum enzyme loading. The optimum concentration of *α*-amylase was 90 IU. Finally optimized conditions are summarized in Table 2. Under these optimized conditions the immobilization efficiency reached 96% and the silica nanoparticle immobilized enzyme gave 5.76 IU/mg activity.

### 3.5. Characterization of Immobilized *α*-Amylase

The enzymatic properties, namely, temperature, salt, and pH optima, *K*_*m*_ and *V*_max⁡_ of immobilized enzyme remained unchanged and matched with free *α*-amylase properties as investigated previously [28].

### 3.6. Starch Hydrolysis

**Figure 7:**
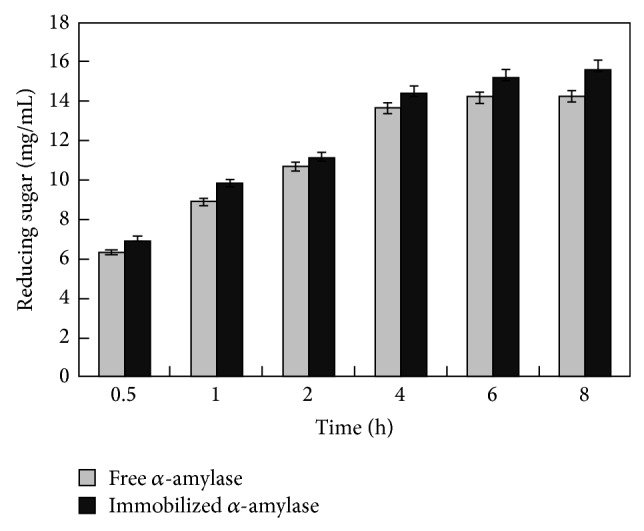
Starch hydrolysis by free and immobilized *α*-amylase. Starch solution (2.0%, w/v) was hydrolyzed by free and immobilized *α*-amylase (2.0 IU/mL) in batch process at pH 7.0 and 45°C in presence of 1% (w/v) NaCl. Samples were withdrawn at different time intervals and reducing sugar was estimated.

Considering the application of immobilized *α*-amylase, the starch hydrolysis was attempted and compared with free enzyme. Results are depicted in Figure 7. The immobilized enzyme hydrolyzed 78% starch in 8 h. The hydrolysis was faster in first 4 h and reached plateau thereafter. The free enzyme exhibited similar trend and hydrolyzed 71% starch in 8 h. Apparently hydrolyzing efficiency of immobilized enzyme was only slightly better as compared to the free enzyme.* Marinobacter* sp. EMB8 *α*-amylase produces maltotriose and maltotetraose upon starch hydrolysis. Maltotriose and maltotetraose rich maltooligosaccharides are more desirable in bread making industries due to their better antistaling properties [28].

### 3.7. Reusability of the Immobilized *α*-Amylase

Reusability potential of immobilized preparation was also explored. The preparation could be used effectively for 2 cycles; thereafter each cycle led to partial loss of activity. It retained 48% of its activity after 8th cycle.

In our opinion, it may not be the loss of activity but it may be loss of nanoparticles suspension going into washing each time. The* Bacillus amyloliquefaciens* *α*-amylase immobilized on polyaniline-assisted silver nanoparticles hydrolyzed only 27% more starch as compared to free enzyme. The preparation could be used 10 times and 20% loss in activity was recorded [24]. Mukherjee et al. [46] reported ~35% starch hydrolysis by* Bacillus alcalophilus* *α*-amylase immobilized on iron-oxide magnetic nanoparticles.

## 4. Conclusions

Halophiles suffer from the drawback of low level of enzyme production, which often limits their applications as source for industrial enzymes. The optimization of culture conditions for achieving maximum amylase production was carried out. Optimization led to about 12-fold increase in the production. The *α*-amylase was immobilized on functionalized silica nanoparticles. Under optimized conditions, 96% immobilization efficiency was obtained. Although the properties of immobilized enzyme remained the same as that of native, it gained in terms of reusability in repeated cycles. The preparation was used for hydrolysis of starch. It is worthwhile mentioning that highlight of the enzyme was to make maltotriose and maltotetraose rich oligosaccharides. Such maltooligosaccharides are highly desirable for application in baking industries due to better antistaling properties.

## Figures and Tables

**Table 1:** Immobilization of *α*-amylase by adsorption and covalent coupling via glutaraldehyde ^*^.

Immobilization procedure	Immobilized *α*-amylase activity (IU)	Unbound *α*-amylase activity (IU)	Immobilization efficiency (%)
Adsorption	16	116	12
Covalent linkage	52	9	40

^*^Total 130 IU *α*-amylase was used for immobilization.

**Table 2:** Optimized conditions for *α*-amylase immobilization on silica nanoparticle.

Parameters varied	Optimized conditions
Crosslinker for functionalization	Glutaraldehyde (0.5%, v/v)
Protectant	Starch (0.5%, w/v)
Amount of functionalized nanoparticles	15 mg
*α*-Amylase loading	90 IU
